# Supplementary material for: Multi-drug resistant bacteria predict mortality in bloodstream infection in a tertiary setting in Tanzania
Source: PLoS One. 2020 Mar 4;15(3):e0220424. doi: 10.1371/journal.pone.0220424 (PMC7055912; doi:10.1371/journal.pone.0220424)
Supplement: Attachment — Submitted filename: Response to reviewer .docx [file pone.0220424.s002.docx]

**Response to editor**

As raised by reviewer #1 there are areas of data detailed within the results which were not addressed within the discussion or conclusions this needs to be resolved. There is also discussion that the subjects were receiving antibiotics (or at least some were).  The authors need to include this very important data into their data tables and analysis.  Specify which antibiotics were being administered and their duration of treatment then analyze to see if there is a direct correlation between the mdr found and the pre-treatment instead of just suggesting this to be a possibility.  The authors also need to include precise cause of death for all subjects, both the positive and the negative blood culture results.  Simply because the blood culture grew that may not have been the underlying cause death. Perhaps there was trauma which precipitated mortality and the bsi was simply secondary to the trauma.

**Response**

Thank you for your valuable comments to improve our manuscript. We have tried to discuss all key findings in our discussion as suggested. All comment on important results missing narrative in text, these has been addressed accordingly.

History of antibiotic use has been added on table 1 and new table 2. It was difficult to get information on exact duration of treatment of these antibiotics, but analysis has been done looking at correlation of antibiotic use prior to culture and isolation of multi-drug resistant bacteria (Table 2) and explained in result section.We have also included analysis of underlying diseases on prediction of mortality on table 4.

**Response to reviewer**

**Reviewer #1**

**Comment**

The manuscript describes a cross-sectional retrospective analysis of bloodstream infections in a limited resource setting with the aim of examining the predictors of mortality of patients with BSI. As the authors indicate, there have been previous similar studies performed at the same setting therefore this work is not particularly original. However, given the limited data available on clinical outcomes of patients with BSI in limited resource settings the study is noteworthy to the scientific community. In general, the publication could benefit from revision of the language to improve the grammar and readability. I suggest the authors work with a copyeditor, as this would be beneficial to the readers. In addition, the use of common clinical microbiology nomenclature in manuscripts is not consistent throughout the manuscript and could be revised, for example hyphenating at times but not others.

R**esponse**

We appreciate for a comment, and we have addressed microbiology nomenclature thought the manuscript and hyphenating has been addressed as well. study.

**Comment**
Methods: Manufacturer name of branded reagents ie, Staphaurex, Strep grouping kit etc. should be included. If a software application was used to perform the statistical analysis it should be included.

**Response:**

Thank you for the comment. We have included the manufacturers of the reagents as suggested line 104 and 105. Also, the software application used has been added, line 128.

**Comment**
The Abstract references Proteus mirabilis, however results for P. mirabilis and A. baumannii are both excluded from any statements in the Results section even though they show high levels of resistance to the antimicrobials in Table 2 and may have had an effect on the mortality metrics. Whether their impact on BSI and mortality is unlikely due to a low N is unclear as they are not discussed in the text.

**Response**

We thank you for this valuable comment, we have added narrative in our text on resistant pattern for Proteus mirabilis and Acinetobacter baummannii, line 192 – 193.

**Comment**
In addition, whether any trends exist by individual bacterial species is not clear given that the organisms are aggregated by type GNR or GPC. The publication would benefit from an analysis to sub stratify by bacteria or at minimum Enterobacteriaceae vs. non-Enterobacteriaceae. If this analysis was performed and there was no significant difference between the groups this should be indicated in the manuscript.

**Response**

Due to small number of individual bacteria stratifications did not bring any significant differences. However, we sub stratified to Enterobacteriaceae and non-Enterobacteriaceae. Significant finding was observed on univariate analysis for isolation of multi-drug resistant bacteria Table 2 and line 183 – 185, but not on mortality table 4.

**Comment**
Figure 1 includes Salmonella typhi which is also never addressed in the results.

**Response**

*Salmonella Typhi* has been included in result line 174 and on table 3.

**Comment**
Consider condensing Table 1 and placing the percentage in parentheses after the frequency value for ease of reading

**Response**

Suggestion accepted and included in table 1

**Comment**
Though the authors indicate some limitations of the study, the impact of such limitations on the data and conclusions is not thoroughly critiqued

**Response**

We have highlighted impact of our limitation on data interpretation and conclusion, line 261 – 272

**Comment**
The study was performed in April-May 2018. Given the short duration, the authors should address whether there is any seasonality that could may have affected prevalence or any other associated limitations.

**Response**

Thank you for comment Line, we have highlighted this in our discussion line 268 - 272

**Comment**
Lines 255-264: the authors highlight the impact of antimicrobial stewardship benefits for BSI treatment however do not cite any references. There are ample publications on Antimicrobial stewardship and BSIs that should be referenced.

**Response**

Citations have been added, line 279 – 280.

**Reviewer #2:**

**Comment**

1.The manuscript is written mostly in clear, standard English, but need some revision with regards to the use of language, sentence constructs, exclusion of words like "the" where it is actually required.
**Response**

Thank you, we have done our best. The manuscript was edited for language by a copy editor

**Comment**
2. Some sentences could be revised to prevent ambiguity (e.g. Line 214: Patients aged more than 14 years suspected of BSI were.......").

**Response**

Line 214 has been revised, now line 229 - 231

**Comment**
3. Capitalization of some words needs to be corrected (Carbapenemases in middle of a sentence should be carbapenemases, "figure 1: in line 176 should be Figure 1, etc.). Another example is "Sixty" in line 180.

**Response**

Capitalization of some words have been worked on thought the document. For example, Figure 1 as seen in line 176 and line 183 Sixty has been corrected

**Comment**
4. Line 215: the authors indicated that certain patients were at high risk of dying compared to those admitted at medical ward. Is that the intended word or did they mean "higher/increased risk"?

**Response**

Thank you, suggestion has been accepted we meant higher/increased risk, now line 231 – 233.

**Comment**
5. Line 183: Remarkably, 46% of Pseudomonas aeruginosa were resistance to meropenem; furthermore, 27% (3/11) of them were Carbapenemase producing pathogens.
“resistance” should be “resistant”; ‘carbapenemase” instead of “Carbapenemase”.

**Response**

Correction have been made, Line 187– 189

**Comment**
6. Table 2 needs some formatting, for example it might be better if the abbreviations of the antibiotics are in one row (not one letter of an abbreviation in a next row).
“Bacteria species” (in row 1 of the table) should be “Bacterial species”
Consistency is needed: all bacteria mentioned in Table 2 denoted with one letter abbreviation (e.g. S. aureus), except for Proteus mirabilis. As this organism were mentioned earlier in manuscript, please change to P.mirabilis, to be consistent with rest of table.
Correction**:** “Percent of bacteria resisted” – suggested change: “Percentage resistance”.

**Response**

All suggestions have been accepted, now it is table 3.

**Comment**
7. Table 3: The number of specimens included in study, based on the suspicion of BSI, equals 402. Yet the number of males and females in Table 3= 352 (117, 235) - should 117 be 167 perhaps?

**Response**

Thank you for noting this, it was typo error, now it has been addressed. Table 4

**Comment**
According to Table3, 46 patients had culture-confirmed BSI, and in line173 it is stated that 46 pathogens were isolated. Yet 48 organisms were isolated (36 GNB,12 GPC) according to Table 3. If this is not an error, I assume one or two patients had a polymicrobial infection. If this assumption is correctly did the patient(s) with more than one organism also succumb to the infection(s), and which combination of organisms was isolated? The number of cases studied here obviously is little, but as our knowledge of polymicrobial infections grow (and the impact on patient outcome), this type of information is valuable. If the number was not a typo error, perhaps mention the specific organisms and patient outcome.

**Response**

There were no polymicrobial infection. This was an error and has been addressed on table 4.

**Comment**
8. The authors discussed that "the hospital need to intensify infection prevention and control program to limit the spread of BSI and its associated mortality". While this statement is true for health care facilities in general, this specific paper did not look at any IPC practices and only briefly mentioned that all patients in this study received antibiotics prior to sampling. No IPC practices (or observed lack) were reported here, neither were studies done to determine if organisms, such as the ESBLs, were identical and spreading in the same facility. Perhaps revise the sentence to indicate that (1) failure of IPC COULD be one contributing factor in the spread of BSI infections in the specific setting, and (2) local IPC practices must be scrutinized to determine which areas needs to be implemented or improved.

**Response**

Thank you for comment, we have omitted the sentence in our discussion.

**Comment**
Secondly, the authors mentioned that "Persistent of high rates of resistance at our setting could be accounted by increasingly empiric use of antibiotics; in most cases imprudent use of antibiotics.". Again, while this is most probably true, we did not see the data from this healthcare facility (not reported in this study) to substantiate this. The alternative should also be considered, that persons could be colonized by resistant bacteria due to antibiotic exposure prior to hospitalization: Increasingly, we see publications of data indicating that outpatient management of UTIs (for example) also increase the risk of ESBL infections. This aspect (antibiotic use outside of health facilities) is neglected in many studies, though much needed especially in settings where antibiotic stewardship is neglected. Perhaps the authors could include a sentence to suggest that evaluation/audit of antibiotic use outside of health facilities (including illegal acquisition of antibiotics without prescription) is also needed, to determine risk factors for future drug resistant infections.

**Response**

In table 1, we have included antibiotics prescribed to these patients before collection of blood. Also, to support our argument we have added table 2, were we found antibiotic use prior to culture had correlated to isolation of MDR bacteria on univariate analysis. In addition, all comments have been accepted line 311 – 315.

**Comment**
9. Regarding the culture negative BSIs: the authors noted that all samples were collected from patients already on antibiotics ("All patients included in the study had used antibiotics prior to blood culture, which might explain the observed low rate of BSI"). This is problematic, as this affects the recovery of organisms. While this might explain the low rate of BSIs, it is reasonable to assume that a fraction of the negative cultures might in fact have been false negatives. Another group of organisms also excluded by this study is anaerobic bacteria (although these usually are low in number, compared to the aerobes).

**Response**

We appreciate for suggestions and have been included in discussion line 263 - 265
