# Supplementary material for: Multi-drug resistant bacteria predict mortality in bloodstream infection in a tertiary setting in Tanzania
Source: PLoS One. 2020 Mar 4;15(3):e0220424. doi: 10.1371/journal.pone.0220424 (PMC7055912; doi:10.1371/journal.pone.0220424)
Supplement: Attachment — Submitted filename: Response to reviewers_28_01_2020.docx [file pone.0220424.s003.docx]

**Response to reviewers**

**Reviewer #1**

**Comment**

The major revisions have been adequately addressed however minor revisions to the writing are still necessary. Several examples are outlined below of modifications that can improve the writing if applied to the entire manuscript.

R**esponse**

We appreciate for a comment, and we have addressed all examples outlined below and other parts

**Comment**

Line 25, Line 51 and Table Titles “Blood-stream” vs. “bloodstream” vs “Blood stream” should be modified to ensure consistent use of one version throughout the manuscript.

**Response**

We have the comment and used one version `Bloodstream` throughout the manuscript.

**Comment**
Also line 30 and 82 “in-patient” vs “inpatient” should be modified to ensure consistency.

**Response**

We have addressed the comment and ensured consistency in using one version “inpatient” throughout the manuscript

**Comment**
Line 30 “all inpatients at THE clinical”

**Response**

The word the has been added in line 31

**Comment**
Line 39, 293, 337 “resistant” not “resistance”

**Response**

We have addressed the comment throughout the manuscript, for example line 40, 367 etc

**Comment**
Line 39 and Line 45…multi-drug resistant vs Multi-drug resistant… Can be capitalized on line 39 to highlight the use of an abbreviation (MDR) in the remainder of the manuscript. Also, for consistency, MDR can be used in lieu of “multi-drug resistant” throughout the remainder of the manuscript once define, ie line 236, 244, 293 etc.

**Response**

Thank you for comment, we have addressed the comment and abbreviated MDR on its first use. Also maintained consistency on use of MDR throughout the manuscript.

**Comment**
Line 41 “Carbapenemase production” …remove the “s”

**Response**

S has been removed as proposed line 42.

**Comment**
Line 176 capitalize Candida and italicize both Candida and albicans

**Response**

The comment has been addressed line 188

**Comment**
Line 189 Piperacillin should be lowercase

**Response**

The comment has been addresses in line 209

**Comment**
Line 190-193 after the first use of the full genus and species the genus can be abbreviated. For example, line 190 Escherichia coli can be abbreviated to E. coli throughout the remainder of the manuscript as it has been defined on line 122. All other organisms should be modified for consistency.

**Response**

Thank you for comment, we have managed to abbreviation for only Escherichia coli, but the rest we have maintained full names of genus and species. This could make easy readability of our work.

**Comment**
Line 174 and Table 3 typhi should be capitalized and not italicized

**Response**

Thank you, the comment has been addressed in line 186

**Comment**
Line 235 “admitted WITH other underlying diseases”

**Response**

With has been added in line 260.

**Comment**
Line 260 Coagulase should be lowercase

Response

The comment has been addressed in line 295

**Comment**
Varied use of “in” or “at” for admission to the wards. Suggest modifying to “to the”…for example Lines 242 and 274-275… “admitted to the ICU” would allow for easier reading. Several other examples exist in the manuscript that should be modified as such.

**Response**

We appreciate for the comment, modification has been made throughout the manuscript. For example, from line 256 to 269.

**Comment**
Figure 1. Be consistent with Genus and Species or Abbreviation and species for all organisms

**Response**

We have made modification and used the full name of genus and species

**Reviewer #2:**

**Comment**

The authors made various corrections and improvements to the manuscript. This paper will be a valuable contribution to our knowledge of BSIs, associated risk factors, etc. on the continent. It potentially could be used to improve on future study designs (especially to account for factors one has no control over (antibiotic use before hospitalization, lack of stewardship in some settings etc).

**Response**
Thank you for the comment and will look forward on designing other studies taking account for factors highlighted.
**Comment**
Two spelling errors still present: The word "malignant" is incorrectly spelled as “Malginant” in Tables 2 and 4.

**Response**

This has been addressed in table 1 and 4

**Comment**
The authors need to pay attention to the references, please modify the abbreviations of journal names for the sake of uniformity (Some abbreviations are not written with the first letter as a capital letter etc). Correct Example: J Clin Gastroenterol. A number of references not abbreviated and not with same format. Examples of incorrect citation: South African medical journal = Suid-Afrikaanse tydskrif vir geneeskunde; also "International journal of biological and medical research."

**Response**

Thank you, we have noted that and we written correct abbreviation throughout the references
